# Biopolymer encapsulation for improved probiotic delivery: Advancements and challenges

**DOI:** 10.3934/microbiol.2024043

**Published:** 2024-11-15

**Authors:** Srirengaraj Vijayaram, Reshma Sinha, Caterina Faggio, Einar Ringø, Chi-Chung Chou

**Affiliations:** 1 Department of Veterinary Medicine, College of Veterinary Medicine, National Chung-Hsing University, 145 Xingda Rd. Taichung, 40227, Taiwan; 2 Department of Animal Sciences, School of Life Sciences, Central University of Himachal Pradesh, Kangra, 176206, India; 3 Department of Chemical, Biological, Pharmaceutical and Environmental Sciences, University of Messina, Viale Ferdinando Stagno d'Alcontres, 31, 98166 S. Agata-Messina, Italy; 4 Norwegian College of Fishery Science, Faculty of Bioscience, Fisheries, and Economics, UiT the Arctic University of Norway, Tromsø, 9037, Norway

**Keywords:** biomedical, biopolymer, encapsulation, functional food, probiotics, viability

## Abstract

Probiotics, known for their health benefits as living microorganisms, hold significant importance across various fields, including agriculture, aquaculture, nutraceuticals, and pharmaceuticals. Optimal delivery and storage of probiotic cells are essential to maximize their effectiveness. Biopolymers, derived from living sources, plants, animals, and microbes, offer a natural solution to enhance probiotic capabilities and they possess distinctive qualities such as stability, flexibility, biocompatibility, sustainability, biodegradability, and antibacterial properties, making them ideal for probiotic applications. These characteristics create optimal environments for the swift and precisely targeted delivery of probiotic cells that surpass the effectiveness of unencapsulated probiotic cells. Various encapsulation techniques using diverse biopolymers are employed for this purpose. These techniques are not limited to spray drying, emulsion, extrusion, spray freeze drying, layer by layer, ionic gelation, complex coacervation, vibration technology, electrospinning, phase separation, sol-gel encapsulation, spray cooling, fluidized, air suspension coating, compression coating, co-crystallization coating, cyclodextrin inclusion, rotating disk, and solvent evaporation methods. This review addresses the latest advancements in probiotic encapsulation materials and techniques, bridging gaps in our understanding of biopolymer-based encapsulation systems. Specifically, we address the limitations of current encapsulation methods in maintaining probiotic viability under extreme environmental conditions and the need for more targeted and efficient delivery mechanisms. Focusing on the interactions between biopolymers and probiotics reveals how customized encapsulation approaches can enhance probiotic stability, survival, and functionality. Through detailed comparative analysis of the effectiveness of various encapsulation methods, we identify key strategies for optimizing probiotic deployment in challenging conditions such as high-temperature processing, acidic environments, and gastrointestinal transit. The findings presented in this review highlight the superior performance of novel encapsulation methods using biopolymer blends and advanced technologies like electrospinning and layer-by-layer assembly, which provide enhanced protection and controlled release of probiotics by offering insights into the development of more robust encapsulation systems that ensure the sustained viability and bioavailability of probiotics, thus advancing their application across multiple industries. In conclusion, this paper provides the foundation for future research to refine encapsulation techniques to overcome the challenges of probiotic delivery in clinical and commercial settings.

## 1. Introduction

The contemporary lifestyle, marked by the extensive embrace of nutritionally inadequate diets and the consumption of “*junk*” food, poses numerous challenges, which include increased prevalence of drug resistance, heightened susceptibility to various infectious diseases, and subsequent overreliance on antibiotics. However, the impacts of immunosuppressive therapy can lead to unfavorable modulation of the intestinal microbiota, contributing to various factors that affect the host's health [1]. The gut microbiota defends the body against invasive bacterial assaults by releasing antimicrobial proteins, including defensins and lysozyme [2],[3]. Both preclinical and clinical data indicate that commercially available probiotic products have the potential to alleviate bowel disorders through the production of key substances such as lactoferrin, cathelicidins, histidines, and IgA antibodies [4]–[6]. When the body's natural defense is compromised, the external supplementation of probiotics, consisting of diverse microorganisms, including yeast and bacteria, becomes imperative [7]. Among probiotics, lactobacilli is favored in mammals, but bifidobacteria, consisting of *Bifidobacterium bifidum*, *B. longum*, *B. animalis*, *B. adolescentis*, *B. breve*, *B. infantis*, and *B. lactis*, are also commonly utilized [7]. In addition, other bacteria and yeasts such as *Bacillus cereus* var. *toyoi*, *Escherichia coli*, *Propionibacterium freudenreichii*, *Saccharomyces cerevisiae*, and *Saccharomyces boulardii* are used as probiotics [7]. Recent research has revealed the multifaceted benefits of probiotics, emphasizing their roles by bolstering the immune function and generating antimicrobial compounds, which mitigate infections and promote host health [8]–[13]. Moreover, antimicrobial proteins have emerged as crucial assets with the escalating threat of antibiotic resistance worldwide. The capability of probiotics to target a broad spectrum of pathogens, including multidrug-resistant strains, positions them as promising candidates for alternative and adjunctive strategies in infection management [14],[15].

The correlation between commercially available probiotics and their effectiveness in alleviating bowel disorders is substantiated by preclinical and clinical data [16]. The evidence underscores the considerable potential of probiotics in fostering gastrointestinal (GI) health. It is intriguing to observe how these minute allies, when harnessed in commercial probiotic products, can significantly contribute to the well-being of the digestive system [16]. Probiotics promote a harmonious balance of microorganisms, thereby enhancing digestive health and potentially averting various health issues—a veritable ‘*Nature's remedy for maintaining a healthy gut ecosystem*’ [17]–[19] or to establish a benevolent intervention aimed at reinstating the healthy equilibrium of bacterial community in the GI tract. The Food and Agriculture Organization/World Health Organization (FAO/WHO) emphasizes the optimal dietary administration of probiotics in the gut, and scientific studies suggested that dietary supplementation with a substantial quantity of probiotic bacteria, around 10^9^ CFU per day, can genuinely augment health benefits in humans [20]. The versatility of probiotics is conspicuous in their availability in various forms, accommodating diverse preferences and lifestyles. Whether encapsulated in convenient capsules, formulated into easy-to-take tablets, or integrated into an array of food products, probiotic cells provide flexibility in how they can be incorporated into daily routines [21]. Several studies have documented the positive impact across various sectors: agriculture, aquaculture, food, nutraceuticals, medicine, pharmaceuticals, and cosmetics [22]–[26]. Despite the notable effectiveness of these beneficial microorganisms, navigating through the digestive system presents considerable challenges, and it is important to ensure their viability by successfully enduring the digestive processes to reach the target organ intact [22]. Moreover, the capacity to be stored under *in vitro* conditions is crucial for practical usability [24],[26]. Encapsulation using biopolymers offers a promising solution to overcome these challenges by providing a protective shield of probiotics. This ensures their survival through the digestive tract and facilitates their storage for optimal effectiveness. Essentially, biopolymers play a crucial role in enhancing the resilience and practicality of probiotics, making them more reliable for consumption and application.

## 2. Aim of the study

We aim to provide an updated overview of encapsulation materials and techniques, focusing on single and combined biopolymer coating methods for probiotics. We aim to explore how encapsulation can enhance shelf-life extension, improve targeted drug delivery, and contribute to the development of bio-functional food products. Additionally, we discuss the potential of coated probiotics in preventing or treating infections. By examining the interactions between biopolymers and probiotics, this study seeks to expand the traditional applications of probiotics and highlight possible synergies that could lead to novel approaches in the field.

## 3. Biopolymer

Biopolymers, synthesized by living organisms, are natural polymers derived from diverse sources, including plants, animals, and microbes [27]–[33], and the biopolymers are widely recognized for their role in the development of biomaterials and drug delivery systems, especially encapsulation of probiotics to enhance their efficacy [27]–[37]. The unique characteristics of biopolymers such as their stability, elasticity, biocompatibility, sustainability, biodegradability, and renewability make them highly versatile for various applications, including food packaging, biomedical engineering, agriculture, environmental remediation, and cosmetics [38]–[44]. Recent studies have highlighted the mechanisms of how biopolymers can encapsulate probiotics, offering protection against harsh environmental conditions like low pH, bile salts, and heat. The encapsulation process involves the formation of a protective barrier around probiotic cells, preventing their degradation in adverse environments such as the stomach. For example, alginate, a common biopolymer, forms a gel matrix in the presence of divalent cations, which creates a physical barrier around the probiotic cells, helping them survive GI transit [45]. Chitosan, another biopolymer, forms polyelectrolyte complexes with alginate, providing additional strength to the encapsulation matrix [46]. Biopolymers offer an eco-friendly degradation process, breaking down into components such as CO_2_, H_2_O, organic macromolecules, and other natural substances [45]. This degradation aligns with their sustainability, minimizing environmental impact and supporting the cyclical nature of materials. Their ability to be naturally reprocessed through biological actions underscores their compatibility with ecosystems, contributing to their eco-friendly profile. Moreover, biopolymers are non-toxic, non-carcinogenic, non-immunogenic, and non-thrombogenic, making them safe for use [47]–[49]. Their carbon-neutral nature further enhances their sustainability. The blending of biopolymers with synthetic materials, such as polyethylene, and polyvinyl alcohol, and plasticizers like sorbitol and glycerine, enables the creation of materials with enhanced mechanical strength, biodegradability, and thermal stability. This combination yields materials that maintain the natural advantages of biopolymers while achieving the improved functionality required for specific applications.

### 3.1. Biopolymer materials used to encapsulate probiotics

The amalgamation of various biopolymers in the encapsulation process generates a synergistic effect that elicits a beneficial response to the encapsulated probiotics [50]. This combined approach serves as a protective shield against enzymatic activity in the GI tract. Concurrently, it facilitates the interaction of probiotics with targeted receptors, ensuring their efficacy in specific areas of the host. This strategic combination amplifies the overall effectiveness of probiotic encapsulation, rendering it a promising approach across diverse applications [50]. Various polymeric materials, including alginate, chitosan, pectin, starch, Arabic gum, xanthan gum, gelatine fat, and glyceride derivatives, can be employed for encapsulation purposes [51]. The diverse range of these materials provides flexibility in the encapsulation processes to meet specific requirements and optimizes the protective properties of the encapsulated substances (Table 1). Biopolymer proteins are derived from animal sources, such as whey proteins, gelatin, and casein, as well as from plant sources, including soy proteins, pea proteins, and cereal proteins. These natural polymers offer several advantages, such as biodegradability, biocompatibility, good amphiphilic activity, and properties that enhance efficiency, like water solubility, emulsifying, and foaming ability. These attributes make biopolymers versatile materials with applications across various industries, ranging from food and pharmaceuticals to cosmetics [51]. Biopolymers offer several advantages, including their ability to dissolve in water, low viscosity even at high concentrations, and capability to facilitate separation processes, such as proteins. The unique combination of properties makes them valuable tools for the development of innovative and sustainable solutions [51].

Table 1 presents an overview of biopolymer materials used, encapsulation techniques, and benefits.

**Table 1.** Types of biopolymer materials, encapsulation techniques, and their benefits.

Biopolymers	Encapsulated probiotics	Encapsulation technique	Improvements/advantages	References
Alginate and gelatin	*Lactobacillus rhamnosus*	Extrusion	Viability in beads at 10^5^ CFU/g after 4 months (initial value 10^9^ CFU/g).	[52]
Alginate	*Lactococcus lactis* spp. *cremoris*	Extrusion	Survived in the intestinal fluid for over 240 min.	[53]
Alginate	*Bifidobacterium pseudocatenulatum*	Extrusion	Improved stability in simulated intestinal fluid (5.6 log10 CFU/g) have.	[54]
Alginate	*Staphylococcus succinus Enterococcus fecium*	Extrusion	Enhanced viability (98.75–88.75%) in simulated gastric fluids. Maintained survival after 30 days of storage at 4 °C and decreased from 8.1 log CFU/mL to 7.9 log CFU/mL in 7 days.	[55]
Alginate and milk	*Lactobacillus bulgaricus*	Extrusion	Enhanced viability in acidic conditions at pH 2.0 after one month of storage at 4 °C.	[56]
Alginate and starch	*Lactobacillus fermentum*	Lyophilization	Significantly higher survival rate (30% higher).	[57]
Alginate, chitosan, and locust beam	*L. rhamnosus*	Freeze-drying	Increased stress and thermos-tolerance in encapsulated probiotic bacteria.	[58]
Alginate and chitosan	*Saccharomyces cerevisiae Y235*	Emulsification	Improved stability (log 6.29 CFUg^−1^) in simulated gastric fluid and simulated intestinal fluid.	[59]
Alginate and chitosan	*B. pseudocatenulatum*	Extrusion	Enhanced viability rate (95%) in simulated intestinal fluid.	[60]
Alginate and chitosan	*Bifidobacterium breve*	Layer-by-layer	Enhanced viability (95%) in *in vitro* gastric condition.	[61]
Alginate, starch, and chitosan	*Lactobacillus acidophilus*	Extrusion	Increased stability in the extrusion technique freeze-dried form by up to log 6.35 CFU g^−1^ for 135 days.	[62]
Alginate, chitosan, and xanthan gum	*Lactobacillus plantarum*	Extrusion	Higher survival (95%) in simulated intestinal fluid and storage stability for 4 weeks at 4 °C.	[63]
Chitosan, agar, and gelatin	*L. plantarum*	Emulsification	No loss of cell concentration after 2 h incubation in gastric fluid and intestinal fluid.	[64]
Chitosan and alginate	Vaccine with *L. plantarum*	Extrusion	Encapsulation of probiotic bacteria used in oral vaccine against spring viremia of carp virus.	[65]
Chitosan and xanthan gum	*Pediococcus acidilactici*	Extrusion	Maintained cell viability for 8 h in the gastrointestinal (GI) fluid with maximum release occurring after 24 h.	[66]
Chitosan and alginate	*B. breve*	Extrusion	Higher survival (95%) in simulated intestinal fluid and storage stability for 4 weeks at 4 °C.	[67]
Chitosan and alginate	*Lactobacillus reuteri* DSM 17938	Vibration technology	Increased resistance in GI stress conditions.	[68],[69]
Chitosan and alginate	Bacteria strain 4.1.Z *(B. amyloliquefaciens, B. subtilis*, and *B. methylotrophicus)*	Vibration and extrusion	Maintained viability (10^6^–10^7^ CFU/g) for about two months under refrigeration conditions.	[70]
Chitosan and alginate	*L. reuteri* KUB-AC5	Emulsification	More stable with a reduction of 1 log CFU/mL after 180 min at pH 1.8.	[71],[72]
Chitosan and hydrochloride alginate	*Bacillus licheniformis*	Orifice-polymerization method	Protected by the chitosan coating for 1 h in simulated GI fluid (pH 2) and 4 h in simulated intestinal fluid (pH 6).	[73]
Rice starch	*L. casei*, *L. brevis*, and *L. plantarum*	Extrusion	Provided more stability in GI and extreme room temperature for 30 days (~3.5 × 10^9^ CFU/mL).	[74]
Starch and pectin	*L. plantarum*	Extrusion	Maintained 5.15 and 6.67 Log CFU/g with pectin and pectin/starch hydrogel, in gastric fluid and simulated intestinal fluid for 2 h.	[75]
Starch from corn and rice	*L. plantarum*	Freeze-drying	Maintained thermal stability and integrity for 35 min at 55 °C.	[76]
Starch, alginate, chitosan, and inulin	*L. casei* and *Bifidobacterium bifidum*	Emulsification	Increased viability when subjected to simulated gastric conditions for 120 min.	[77]
Starch	*Lactobacillus paracasei*	Electrospinning	Maintained stability under 4 °C and 37 °C for 21 days under storage conditions.	[78]
Maize starch, maltodextrin, and gum Arabic	*L. acidophilus*	Spray-drying	Maintained stability under storage conditions after 30 days.	[79]
Cassava starch and alginate	*L. brevis*	Emulsification	Increased efficiency by 95% in GI conditions.	[80]
Cellulose and pectin	Lactic acid bacteria	High-pressure Microfluid-isation	Showed resistance in acid medium condition.	[81]
CMC and inulin	*L. plantarum*	Casting	Significantly increased the viability (36%) during storage time	[82]
Cellulose, alginate, starch, and lecithin	*Lactobacillus rhamnosus*	Extrusion	Increased viability by 37% in GI fluid at 25 and 4 °C.	[83]
CMC and rice bran	*Lactobacillus reuteri*	Emulsification	Decreased cell viability after heat exposure (85 °C, 25 s).	[84]

Abbreviations: CMC-carboxymethyl cellulose; CFU/g-colony-forming unit per gram; and CFU/mL-colony-forming unit per milliliter.

#### 3.1.1. Algae-derived alginate for probiotic encapsulation

Algae, particularly brown seaweed, serves as a rich source of alginate, a naturally derived polymer widely utilized in the encapsulation of probiotic cells [85] and is a favored material for encapsulation due to its unique properties, including biocompatibility, versatility, and ability to form gel-like structures. Brown seaweeds, such as *Laminaria hyperborea* and *Ascophyllum nodosum*, are the primary sources of alginate. Alginate is a linear anionic polysaccharide composed of (1–4)-linked copolymers of α-l-guluronic acid (G) and β-d-mannuronic acid (M), with variations in the M and G residues occurring during the polymerization process [85].

The extraction process involves harvesting and processing the seaweed to obtain alginate, done through a series of alkaline treatments followed by precipitation and purification steps [86]. The encapsulation of probiotics with alginate enhances their viability in comparison to free-growing cells [86] and creates a protective matrix around the probiotic cells, effectively shielding them from acidic environments in the stomach and enzymatic digestion in the GI tract. This increased protection contributes to the improved survival and functionality of probiotics, making alginate encapsulation a valuable strategy for enhancing their efficacy [86].

Alginate encapsulation also enables the incorporation of probiotics into a wide range of food products, including dairy, beverages, and functional foods, enhancing their nutritional value and health benefits. Additionally, alginate-based encapsulation offers controlled-release formulations for probiotic supplements, ensuring precise dosing and therapeutic efficacy in pharmaceutical applications [87]. Algae-derived alginate serves as a valuable encapsulation material for probiotics, offering numerous advantages in terms of stability, delivery, and efficacy. Its widespread application in various industries underscores its importance as a versatile and biocompatible polymer for probiotic encapsulation. Alginate polymer is used to create hydrogel bead layers that serve as a protective shield for *Bifidobacterium breve* when exposed to gastric juice under low pH conditions [87]. These microbeads effectively preserve the vigor and vitality of the encapsulated probiotic despite challenging environmental conditions [46]. However, the presence of cations such as calcium, sodium, and magnesium, which are generated during probiotic fermentation, can adversely affect the gelling action of alginate, potentially diminishing its effectiveness as a protective matrix [88]. In experiments with *Lactobacillus casei* NCDC-298 encapsulated in alginate at concentrations of 2, 3, or 4%, the probiotic cells were subjected to either low pH (1.5), high bile salt concentrations (1 or 2%), and heat treatment (55, 60, or 65 °C for 20 minutes) and found that the alginate encapsulation significantly enhanced the viability of the probiotic cells under these harsh conditions. Additionally, the encapsulation improved the resilience of the probiotic cells and contributed to the quality of functional food products [87].

Hydrogels, three-dimensional hydrophilic polymer networks that can absorb substantial amounts of water or biological fluids, have gained attention due to their utility in probiotic encapsulation [89]. These biocompatible, biodegradable, and tuneable polymers can be composed of natural and synthetic sources [90], making them highly adaptable for biomedical and food applications. As described by Summonte et al. [91], the formation of hydrogels involves either physical or chemical cross-linking, depending on the material used. Physical hydrogels are stabilized by non-covalent interactions like hydrogen bonding and ionic interactions, while chemical hydrogels require covalent cross-linking. These interactions trap probiotic cells within the hydrogel network, showing enhanced survival under conditions such as low pH or bile salts [92]. Alginate hydrogels, one of the most studied systems for probiotic encapsulation, are formed when alginate chains are cross-linked with divalent cations, such as calcium [93]. This system provides a robust protective matrix for probiotics against acidic conditions, facilitating release in the intestines [94]. Chitosan, derived from chitin, forms hydrogels through ionic interactions and is widely reported [95] to offer improved mechanical stability and antibacterial properties. Its synergy with other polymers like alginate, enhances the encapsulation efficiency of probiotics, providing better protection during gastric transit. According to Srivastava & Choudhury [96] gelatine-based hydrogels are commonly used in food and pharmaceutical industries due to their biocompatibility and gelation properties. Gelatine, in combination with other polymers like chitosan, can improve encapsulation performance by providing a stable matrix for probiotic delivery. Synthetic hydrogels based on polyethylene glycol (PEG) have been explored for controlled release. Patarroyo et al. [97] demonstrated that these hydrogels can be fine-tuned for mechanical strength and degradation rates, although their application in food is limited due to safety concerns.

#### 3.1.2. Chitosan

Chitosan, a polysaccharide derived from chitin, isfound in crustacean shells, insects, algae, and fungi [52]. It is a widely employed biodegradable polymer known for its renewability and cost-effectiveness, making it a viable and sustainable material [85]. Another algal polymer, carrageenan biopolymer, is of interest in both research and the food sector [88], as the microcapsules developed with this biopolymer significantly enhance the viability of probiotic cells, offering improved protection against stressful conditions [98]. The chitosan-coated material not only increased the surface area but also enhanced the bio-adhesion potential while maintaining the viability and growth kinetics of probiotics without adverse effects [99].

#### 3.1.3. Gelatine

Gelatine, being a soluble protein and relatively more cost-effective than other biopolymers, is one of the most used hydrocolloids in the food industry as it also acts as a thermal reversible gelling agent employed in the encapsulation of probiotic cells. Gelatine is extracted from various sources such as pig skin and bones (46% and 23.1%), cowhide (29.4%), and fish (1.5%), and is produced after partial hydrolysis of collagen [85],[100]. Additionally, gelatine is a promising candidate for combination with anionic gel-forming polysaccharides, such as gellan gum. This combination offers potential to create formulations with enhanced properties, catering to specific requirements in various applications [93]. At pH below 6, gelatine undergoes positive charging and establishes a strong affinity with negatively charged gellan gum. These hydrocolloids are mixable at a pH lower than 6. Higher concentrations of gelatine can also be employed for the encapsulation of *Lactobacillus lactis* through cross-linking with toluene-2,4-diisocyanate [101].

#### 3.1.4. Pectin

**Figure 1.**
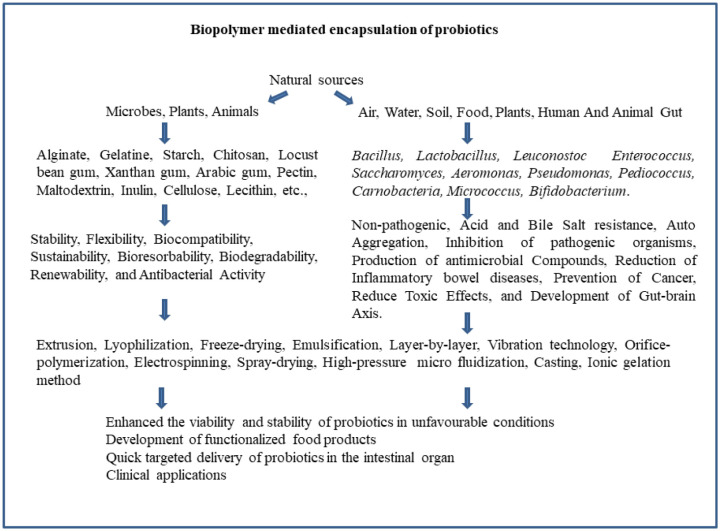
Biopolymer encapsulation of probiotics.

Pectin polysaccharide is a linear and anionic molecule derived from apple pulp and citrus fruits. It is a high molar mass heteropolysaccharide with at least 65% wt of α-(1–4)-d-galacturonic acid-based units. These units are presented either as free acid, salt forms (sodium, calcium, ammonium, and potassium), esterified with methanol, or as acid amidated pectins. The versatile nature of pectin allows for various modifications, enabling its application in a range of industries, including food and pharmaceuticals [102]. Pectin is classified based on its degree of methoxylation and is commonly utilized by the food sector due to its impressive gelling power and is a superior material for the encapsulation process due to easy degradation by microbiota, thus facilitating controlled delivery of encapsulated compounds. This characteristic makes pectin a valuable choice in applications where sustained release or targeted delivery is desired [85]. Encapsulation materials, including pectin, alginate, and whey proteins, are suitable for encapsulating *Bifidobacterium* cells in a mixed gel [103]. Two types of capsules can be prepared, the one with an additional membrane and the one with beads lacking an extra membrane. Studies evaluating the importance of the membrane on the viability of probiotic cells revealed that beads with extra membranes exhibit increased survival (log 4–7) compared to cells in beads without membranes (log 2–3) at gastric pH (pH 2.5) and in 2 and 4% bile salt solutions for 1 and 3 hours. This underscores the protective role of the extra membrane in enhancing the survival of probiotic cells under challenging conditions [104]–[112]. The encapsulation process may entail the coating of single or combined biopolymer materials and it can range from micro to nano-encapsulation of probiotic strains. Various techniques are employed in this process, including spray drying, extrusion, and freeze-drying, as shown in Figure 1. These methods contribute to the development of encapsulated probiotic products with enhanced stability, viability and targeted delivery [113]–[116]. Biopolymer microcapsules, those made from alginate or a combination of alginate and starch, as well as materials like xanthan and κ-carrageenan, offer effective protection to *L. reuteri* cells against acidic conditions. These microcapsules contribute to the improvement of the viability and stability of the probiotic cells, enhancing their potential for survival in challenging environments, particularly in acidic gastric conditions. The use of these encapsulation materials is a valuable strategy for maintaining the functionality of probiotics during their passage through the digestive system [117]–[119]. The utilization of skim milk particles is reported to enhance the viability of probiotics, including strains such as *Lactobacillus acidophilus* La-5 and *B. breve*. This approach promotes the survival and stability of probiotic cells, potentially by providing a protective matrix or support system for these microorganisms. The use of skim milk particles represents a novel strategy to improve the efficacy of probiotics during their transit through the digestive system [120].

## 4. Encapsulation techniques, efficiency, and storage stability

Encapsulation of probiotics is a vital process in the food and nutraceutical industries, enhancing the stability, viability, and controlled release of beneficial microorganisms. Encapsulation methods are broadly classified into chemical and mechanical/physical categories, each offering distinct advantages and limitations. Chemical encapsulation techniques involve chemical reactions to form encapsulating materials, with coacervation and co-crystallization being prominent examples. These methods offer precise control during the encapsulation process and enable the creation of materials with tailored properties. The advantage of chemical methods is their ability to provide controlled release profiles, which can protect probiotics until they reach specific locations in the GI tract [120]. Additionally, chemical methods enable the customization of encapsulating materials to meet the specific needs of different probiotic strains and applications [121]. These methods can, however, be complex and costly due to the need for precise control over reaction conditions [122]. Scaling up chemical processes from the laboratory to industrial scale may also present challenges [123]. Mechanical or physical methods, including spray drying, freeze-drying, and extrusion coating, utilize physical processes for encapsulation. These methods are generally simpler and more cost-effective compared to chemical techniques [124]. They offer versatility and can be applied to various substances, including probiotics and other bioactive compounds [125]. Despite their advantages, mechanical methods may provide less control over release profiles compared to chemical methods, potentially resulting in less tailored delivery [126]. Additionally, some methods, like spray drying, can subject probiotics to physical stress, affecting their viability if not properly managed [127]. Future advancements in encapsulation technology are expected to focus on improving the efficiency and specificity of the processes. Innovations may include the development of novel biopolymers with enhanced properties for controlled release and the integration of advanced characterization techniques to optimize encapsulation outcomes [128]. Combining different encapsulation methods could also increase the strengths of each approach, addressing limitations and improving the delivery and efficacy of probiotics and other bioactive substances. These developments hold promise for enhancing the functionality and health benefits of encapsulated products in various industries, including food, pharmaceuticals, and nutraceuticals.

### 4.1. Human-related information

Biopolymer-encapsulated probiotics have garnered considerable interest for their potential benefits to human health, particularly in enhancing gut microbiota stability and digestive health. The encapsulation of probiotics using biopolymers such as alginate, chitosan, or gelatin offers a protective barrier that shields probiotics from harsh GI conditions, including acidic environments and bile salts [129]. This protection ensures that many viable probiotic cells reach the intestines, where they can exert their beneficial effects. Enhanced survival and viability of probiotics through encapsulation directly contribute to improved gut health by maintaining a balanced microbiota and supporting the intestinal epithelial barrier. This balance is crucial for preventing dysbiosis, a condition associated with various GI disorders [130]. Additionally, encapsulated probiotics have been shown to enhance immune function, potentially reducing the incidence of infections and inflammation [9]. Beyond GI benefits, biopolymer-encapsulated probiotics also have implications for metabolic health. For instance, probiotics can influence metabolic processes, such as glucose metabolism and lipid profiles, which are critical for managing conditions like diabetes and obesity [131]. Encapsulation enhances the stability and delivery of probiotics, thereby amplifying their effects on metabolic health. Furthermore, encapsulated probiotics have shown promise in enhancing the effectiveness of probiotics in fermented foods and dietary supplements. This improved efficacy can contribute to better health outcomes in areas such as reducing GI discomfort and supporting overall well-being [132].

### 4.2. Fish-related information

Biopolymer-encapsulated probiotics have shown significant potential in improving fish health through multiple mechanisms. The use of encapsulation techniques, such as those involving alginate, chitosan, or other biopolymers, enhances the survival and stability of probiotics. These techniques protect probiotics from environmental stresses during storage and processing, ensuring that a high number of viable probiotic cells reach the fish's digestive tract [8]. Once in the gut, encapsulated probiotics can positively influence gut health by maintaining a balanced beneficial gut microbiota, improving digestion, and enhancing nutrient absorption, which collectively contributes to the fish's overall health and growth performance [1],[133]. Moreover, encapsulated probiotics can enhance disease resistance in fish by boosting the immune system and outcompeting harmful pathogens in the gut. This improved immune response helps protect against infections and diseases [7]. The efficacy of encapsulated probiotics also extends to growth performance, as they facilitate better nutrient utilization and growth rates, which are crucial for the efficiency of aquaculture operations [10]. Additionally, these probiotics help mitigate the adverse effects of environmental stressors by stabilizing the gut microbiome and supporting stress response mechanisms [134].

### 4.3. Spray drying

The encapsulation of probiotics can be achieved through various processes, demonstrating the versatility of encapsulation techniques. Some of these methods include extrusion coating, emulsification, spray drying, spray cooling, spray chilling coacervation, freeze-drying, fluidized bed coating, co-crystallization, multiple microemulsions and liposomes, compression coating, spray coating, and rotational suspension separation. Each technique offers unique advantages and may be selected based on specific requirements, such as the characteristics of the probiotics, the desired release profile, and the intended application of the encapsulated material.

In the realm of biopolymer encapsulation within the food industry, a prominent and extensively employed technique involves amalgamating alginate with various other polymers. Alginate encapsulation, especially when synergized with substances such as chitosan, gelatine, or other biopolymers, stands out as a favored and widely adopted method in food technology [135],[136]. The process involves several key stages in the preparation of biopolymer encapsulation. Initially, a biopolymer solution is meticulously crafted. Subsequently, the core materials are prepared to integrate with the biopolymer matrix. This methodology enhances the stability and viability of probiotic cells, providing robust protection against adverse environmental factors such as lactic and acetic acid, the harsh conditions of the GI tract, and even freezing temperatures. Beyond fortifying the resilience of probiotics, this technique assumes a pivotal role in advancing the development of novel bio-functional foods and nutraceuticals, thereby contributing to the overall enhancement of functional food technologies [119],[137],[138]. Utilizing encapsulation techniques involving alginate or other compatible materials proves instrumental in elevating the survival rates of bifidobacteria, especially in challenging conditions marked by temperature variations and acidity fluctuations during storage. This approach demonstrates the immense potential for effectively delivering viable probiotics to the human GI tract. The enhanced survival capability underpins the promise of utilizing encapsulation as a reliable strategy for ensuring the successful transit of probiotic microorganisms through the harsh digestive environment to exert their beneficial effects in the GI tract [139],[140]. This method proves invaluable in facilitating the targeted release of *L. acidophilus* La-5 within the human gut, particularly through the incorporation into dairy-fermented products. By employing this technique, the controlled and sustained release of *L. acidophilus* La-5 is achieved, ensuring its optimal delivery to the specific GI regions in humans. This application underscores the potential of the technique as a strategic tool for enhancing the effectiveness of probiotic delivery through dairy-based formulations.

### 4.4. Extrusion

This encapsulation method is a pivotal, uncomplicated, cost-effective, and gentle technique. Its simplicity contributes to its cost-effectiveness, rendering it a practical choice for encapsulation processes. The gentle nature of this technique results in reduced cell injuries, leading to a discernible increase in the viability of probiotic cells [67]. The method's effectiveness lies in its ability to delicately encapsulate probiotics, ensuring their integrity and functionality are preserved, thereby enhancing their overall feasibility for various applications. The preparation of a hydrocolloid solution typically entails dissolving hydrocolloid substances in water or other liquid mediums. Hydrocolloids represent a diverse group of polymers characterized by their capacity to form gels or thickened solutions upon dispersion in water. Owing to their exceptional thickening, gelling, and stabilizing properties, these substances find extensive use across diverse industries, such as food, pharmaceuticals, and cosmetics. Their versatile characteristics make hydrocolloids indispensable for various applications, contributing significantly to the texture, consistency, and stability of products in different sectors. In this encapsulation process, a hydrocolloid solution is meticulously prepared and applied to probiotic cells. The solution is then carefully dispensed through a syringe needle or nozzle. The methodology involves the extrusion of a mixture containing probiotics, and the encapsulation occurs as beads through this extrusion process into a CaCl_2_ (calcium chloride) solution. Notably, the temperature plays a crucial role in the dissolution and gelation process of the calcium chloride solution. This is particularly significant in applications like alginate encapsulation, where calcium ions are pivotal in inducing gel formation. The controlled temperature during this stage is essential for regulating the gelation process, ensuring the successful encapsulation of probiotic cells within the hydrocolloid matrix. This temperature-sensitive step is a key factor in determining the efficacy and quality of the encapsulation procedure [141],[142]. In this encapsulation method, various wall materials are employed to encapsulate probiotics, such as lactobacilli and bifidobacteria. The diverse range of wall materials includes alginate, k-carrageenan, a combination of k-carrageenan and locust bean gum, a blend of xanthan and gellan, a mixture of alginate and corn starch, and whey proteins. These materials contribute unique characteristics to the encapsulation process, influencing factors like stability, release properties, and protection of the probiotic cells. This approach allows flexibility in tailoring encapsulation to specific probiotic strains and desired outcomes, showcasing the method's versatility in achieving varied encapsulation objectives [143]. The dimensions of the encapsulation beads in this method are contingent upon the dimensions of the nozzle used and the alignment between the nozzle and the setting bath. The sizes of the beads can exhibit a considerable range, typically spanning from 200 µm to 5 mm. The careful calibration of these parameters is critical for achieving desired bead sizes, ensuring precision and uniformity in the encapsulation process. The ability to control bead dimensions within this specified range enhances the method's versatility, enabling tailored applications across a spectrum of uses and requirements [144]. The use of a composite of xanthan and gellan materials has demonstrated superior stability under gastric conditions when compared to materials like alginate, k-carrageenan, or locust bean gums. The effectiveness of this combination highlights its potential for enhancing the survival of encapsulated substances, such as probiotics, through the challenges of the GI environment. The size and shape of capsules formed during the gelation process, particularly with biopolymer materials like alginate and calcium chloride, are subject to various influencing factors, including the concentrations of the solutions, the viscosity of the biopolymer solution, and the conditions maintained during gelation. The careful control and optimization of these factors play a crucial role in determining the physical characteristics of the capsules, impacting their functionality and effectiveness in delivering encapsulated materials under specific conditions [145].

### 4.5. Emulsification

This method is effective for microencapsulating biopolymers on lactic acid bacteria and various other probiotic microbes [146]. When formulating an encapsulation solution that involves altering the combination of probiotic culture and sodium alginate, a thoughtful approach is essential to achieve the desired characteristics of the encapsulated product. This method offers a versatile platform for tailoring the encapsulation process to specific probiotic strains, *L. acidophilus*, *Bifidobacterium lactis*, and *Lactobacillus plantarum*, ensuring precision in achieving the intended characteristics of the final product. The thoughtful formulation accounts for the unique attributes of the probiotic culture and sodium alginate, optimizing their combination for enhanced microencapsulation results. The liquefied combination is then carefully transferred into vegetable oil, which is pre-dispersed with Tween-80 at 0.2%, serving as an emulsifier. This emulsifier is pivotal in enhancing the overall encapsulation process, promoting efficient distribution and a creamy texture in the resultant mixture. Following this, the beads are effectively fixed to the bottom of the beaker, ensuring a controlled and stable environment for further processing. This step is crucial for maintaining the integrity of the encapsulated material and facilitating subsequent stages of the encapsulation procedure. In this process, a small volume of a cell/polymer slurry is introduced into a larger quantity of vegetable oil, such as sunflower, corn, soy, millet, or light paraffin oil. Through careful agitation and stirring, tiny particles of the water segment within the water/oil emulsion coalesce to form beads of smaller sizes. The water-soluble polymer transforms to become insoluble after the addition of calcium chloride, a cross-linking agent, leading to the creation of gel-like particles in the oil phase. To separate the components, the solution undergoes centrifugation. Subsequently, the beads are incorporated together, and the oil layer is rearranged. This step is crucial for refining the encapsulation process and ensuring the proper arrangement and integrity of the encapsulated material within the oil phase [147]. The ratio of agitation in the combination and the type of emulsifier utilized are critical factors influencing the size and shape of the beads formed. The inclusion of emulsifiers plays a significant role in reducing the size of the beads by lowering the interfacial tension between the water and oil phases. Notably, studies have indicated that employing emulsifiers like Tween 80 and lauryl sulfate mixture can generate beads within a range of 25–35 µm in diameter. The careful control of these factors allows for the precise modulation of bead characteristics, demonstrating the importance of these parameters in tailoring the microencapsulation process for specific outcomes [147]. The size of the beads plays a crucial role in determining the viability of probiotic cells, the metabolic ratio, and the sensory attributes of the final product. It significantly influences the distribution and spreading of excellence throughout the product. The individual importance of viable probiotic cells relies on their successful encapsulation within the beads, ensuring their survival and functionality. Additionally, the metabolic ratio, or the rate of metabolic activity, is influenced by the size of the beads and how efficiently they release the encapsulated probiotics. Moreover, the sensory aspects of the final product, including taste, texture, and overall consumer experience, are impacted by the size and distribution of the microbeads. Achieving an optimal bead size is essential to ensure a harmonious integration of probiotics into the product, enhancing both the product's functional and sensory qualities [148]. While the emulsification method can be relatively costly due to the need for a large quantity of vegetable oil for emulsion formation, it offers notable advantages compared to the extrusion technique. The emulsification method can be readily scaled up, making it more feasible for large-scale production. Additionally, the size of the beads produced through emulsification is significantly smaller, ranging from 25 µm to 2 mm [78]. Furthermore, the emulsion technique provides the flexibility to incorporate fat-soluble acids such as acetic acid into the encapsulation combination when using alginate. This versatility allows for a broader range of formulations, enhancing the potential applications of the method in the encapsulation of various compounds, including probiotics. The combination of scalability and flexibility makes the emulsification method an attractive choice for certain applications despite its associated costs [149].

### 4.6. Pray–freeze-drying

Rutherford et al. [150] examined the encapsulation of freeze-dried probiotics using molten lipids, specifically with a composition of 60–75% stearic acids at 60 °C, employing a spray cooling method to achieve encapsulates with a size ranging from 75 to 300 µm. This innovative technique proves effective in preventing heat-related damage during food processing and concurrently enhances the stability of the final product. By utilizing molten lipids, the encapsulation process protects the probiotics from the adverse effects of heat, ensuring their viability and functionality. The resultant encapsulates offer not only precise size control but also enhance the overall stability of probiotics in diverse food applications. The biopolymer encapsulation process for freeze-dried probiotics is distinguished by its notably short processing time. However, there is limited information regarding the survival of freeze-dried probiotics at the specific temperature of 60 °C. In the microencapsulation process employing the freeze-drying method, the probiotic organisms are separated within a saturated solution of wall materials. This separation is achieved through freezing at lower temperatures, a phenomenon observed through the conversion of frozen water under a vacuum. The freeze-drying method is known for its ability to preserve the integrity of probiotics by removing moisture under controlled conditions. However, the survival of probiotics, especially at higher temperatures like 60 °C, warrants careful consideration and further investigation due to the potential impact of heat on probiotic viability [142],[151]. While the processing stages in biopolymer encapsulation are generally gentle, it's important to note that during the freezing stage, there is a potential risk of loss of cell viability. The maximal loss typically occurs during the hushing stage, specifically at temperatures ranging from −4 °C to −20 °C. This stage can impact the cell membrane, potentially leading to a reduction in cell viability. The sensitivity of cellular components to higher temperatures during freezing is a critical consideration. Elevated temperatures during freezing may adversely affect important cellular components, influencing the overall viability and functionality of the encapsulated material [152]. Therefore, maintaining optimal conditions during the freezing stage is crucial to minimizing potential damage to the cell membrane and preserving the integrity of cellular components [152]. The physical conditions encountered during the drying processes in biopolymer encapsulation can impose additional stresses on the encapsulated cells. The combination of freezing and subsequent drying introduces various challenges that the cells must endure. Freeze-drying involves transitioning the frozen water in the sample directly to a vapor phase under vacuum conditions. This process, known as sublimation, can subject the cells to mechanical stress due to ice crystal formation and removal of water. Additionally, the cells may experience osmotic stress as the surrounding environment changes during the drying process. Careful control and optimization of these drying conditions are essential to mitigate stresses and enhance the viability and stability of the encapsulated cells.

### 4.7. Layer-by-layer

The layer-by-layer (LbL) technique is a comprehensive method in biopolymer encapsulation and systematically addresses a range of challenges, encompassing chemical, physical, and probiotic-specific encapsulation considerations. Through a layering process, alternating materials are deposited on the surface of the probiotic cells, creating a multilayered protective coating. Chemical challenges, such as the compatibility of encapsulation materials, are addressed by carefully selecting and depositing layers to create a tailored encapsulation matrix. Physically, the LbL technique enables precise control over the thickness and composition of each layer, optimizing the overall encapsulation structure. Moreover, the method is adaptable to the specific needs and characteristics of probiotic cells. It provides a versatile platform to address probiotic-specific challenges, ensuring the encapsulation process is fine-tuned to enhance the survival, stability, and functionality of the encapsulated probiotics. The LbL technique, thus, emerges as a sophisticated and versatile approach in the realm of biopolymer encapsulation [153] and is a versatile technique utilized for coating surfaces by sequentially depositing alternating layers of materials, typically driven by electrostatic interactions. This technique is not limited to surface coating but can also be applied to create multilayer coatings on particles or capsules. The LbL approach offers a range of benefits, including enhanced stability, controlled release, and the incorporation of other desired functionalities into the coated structures [153]. By carefully selecting and depositing materials systematically, the LbL technique enables the creation of tailored coatings with precise control over thickness and composition. This level of customization makes it particularly valuable in various applications, such as encapsulation, where the properties of the coating can be fine-tuned to meet specific requirements, ultimately improving the performance and functionality of the coated particles or capsules. Encapsulated probiotics effectively address challenges posed by the GI tract through a defensive mechanism that involves mucus adhesion and growth on intestinal surfaces. The encapsulation process provides a protective layer around the probiotics, enabling them to navigate the harsh conditions of the GI tract more effectively [154]. These encapsulation methods offer positive effects, notably the enhanced survival and targeted delivery of probiotics to the small intestine *in vivo*. The protective encapsulation provides a shield for probiotics, safeguarding them from the harsh conditions encountered during their transit through the digestive system. As a result, the encapsulated probiotics have an increased likelihood of surviving the challenges of the stomach environment and reaching the small intestine in a viable state [155]. In essence, these encapsulation methods play a critical role in optimizing the delivery and functionality of probiotics, offering a more effective means of harnessing their health-promoting benefits *in vivo*.

### 4.8. Electrospinning

The electrospinning technique is a fiber production process that utilizes electric forces to draw fine threads from polymer solutions and involves the application of an electric field to a polymer solution or a combination of polymers. The electric field, along with controlled stretching and other specific conditions during electrospinning, enables the production of nanofibers with diameters in the order of several hundred nanometers. In electrospinning, a polymer solution is subjected to an electric field, causing the formation of a charged jet. The solvent in the jet evaporates as it travels towards a grounded collector, resulting in the solidification of the polymer fibers. This process enables the creation of nanofibers with high surface area and unique properties, making electrospinning a versatile technique widely used in various applications, including tissue engineering, drug delivery, filtration, and other fields where fine fibers with specific characteristics are required. Electrospinning stands out as a unique fiber production process that combines elements from both electrospraying and traditional dry spinning of fibers. The electrospinning process allows to production of fibers with diameters at the nanoscale, providing a high degree of control over the resulting material's structure and properties. This fine-tuned control is particularly valuable in creating materials with enhanced surface area, porosity, and mechanical strength. In materials science, these nanofibers find applications in creating advanced materials with tailored characteristics. In biotechnology and nanotechnology, electrospinning is employed for various purposes, including the development of drug delivery systems, tissue engineering scaffolds, and nanocomposite materials [99]. The versatility of electrospinning makes it a valuable tool for researchers and engineers seeking precise control over the characteristics of fibers in their applications [155]. Using electrospinning-coated techniques to encapsulate nanofibers has significantly extended the shelf life of the encapsulated materials, especially at room temperature (20–25 °C). The electrospinning process enables the formation of nanofiber coatings with a high surface area and allows for precise control over the encapsulation, enhancing material stability and protection. This coating acts as a protective barrier, shielding the encapsulated materials from environmental factors that could compromise their viability [156]. The encapsulation of novel nanofiber mats, comprising a combination of chitosan (CS) and polyvinyl alcohol (PVA) loaded with *Bifidobacterium animalis* subsp. *Lactis* Bb12 and inulin (INU), through the electrospinning technique, involves a sophisticated process [99]. This sophisticated process allows for the precise control of the composition and structure of the nanofiber mats, providing a platform for the efficient encapsulation of probiotics and other bioactive compounds for various applications, particularly in biotechnology and probiotic delivery systems. The scanning electron microscope (SEM) examination has confirmed that the encapsulated nanofiber mats exhibit a diameter range from 117.5 ± 70.6 to 217.6 ± 62.7 nanometers [157]. This analysis provides valuable insights into the physical characteristics and uniformity of the nanofiber mats, crucial for understanding their structural properties. Additionally, a thermal examination of the fibrous mats has been conducted to analyze the heat resistance of probiotic cells within these mats. This examination is particularly significant in assessing the viability and stability of probiotic cells when exposed to heat, which is crucial for applications in dairy and non-dairy foods. Understanding the thermal properties of the encapsulated probiotic-loaded nanofiber mats is essential for ensuring the preservation of probiotic functionality during food processing and storage, contributing to the development of effective probiotic delivery systems in various food matrices. The encapsulation of (CS/PVA)/INU electro-spun fibers has demonstrated a significant increase in the survivability of probiotic cells under simulated gastric juice and intestinal fluids. This outcome suggests a promising potential for these electrons pun fiber mats to protect probiotic cells during the digestive process, ensuring their viability as they reach the GI tract. The electro-spun fiber mats, by providing a protective barrier, have successfully enhanced the survival rate of probiotic cells, which is crucial for their efficacy in promoting health benefits. This finding is particularly relevant in the context of developing functional foods where the delivery and survival of probiotics in the GI tract are essential for their intended effects. The encapsulation approach with CS/PVA/INU electro-spun fibers not only contributes to the improved survivability of probiotic cells but also opens alternative avenues for enhancing the functionality of food products. This research outcome underscores the potential of electrospun fiber mats as a viable strategy for incorporating and preserving probiotics in functional foods [158]–[160]. The electrospinning process involves the application of high voltage to stretch and expand a polymer solution or melted polymer. In this context, electrospinning is utilized to fabricate core-sheath composite nanofibers using starch and alginate. These composite nanofibers serve as the encapsulating material for viable probiotic cells. By leveraging the electrospinning technique, the resulting core-sheath composite nanofibers offer a controlled and protective environment for the encapsulated probiotic cells. This method allows for precise control over the structure and composition of the nanofibers, providing a promising approach for the development of functional materials for probiotic delivery and other applications [115],[161]–[164]. The electrospinning method employing starch format fibers has been shown to enhance the stability and viability of probiotic cells. This process creates a favorable environment for probiotic encapsulation, potentially leading to the development of biotherapeutic agents [159]. The alginate-based electro-spun encapsulation of *Lactobacillus paracasei* KS-199 in fiber mats is a promising strategy for improving the protection, survival, and viability of probiotic cells. This has implications for the development of functional foods and supplements with enhanced probiotic efficacy [160]. Single or dual mixtures of biopolymers are coated by the electro-spun method. The approach defends certain probiotic cell strains in the foodstuff and digestive tract [161]–[163]. Electrospinning-based probiotic delivery systems using nanofibers offer a promising approach for the targeted delivery of probiotics to mucosal surfaces, including oral, nasal, or vaginal surfaces. This approach holds great potential for applications in oral health, nasal health, vaginal health, and other areas where targeted delivery and controlled release of probiotics are beneficial [162]. The electrospinning method indeed offers a solution to enhance the viability of probiotics, particularly by producing nanofibers in the nano and micro ranges. Electrospinning is a powerful technique used for fabricating nanofibers through the application of an electric field. This approach offers a controlled and gentle means of producing probiotic delivery systems, potentially enhancing their viability and functionality [164],[165]. The electrospinning process was utilized to assess the feasibility of lactobacilli and bifidobacteria, through *in vitro* methods [165]. In addition, the nano-encapsulation of electrospinning fiber mats within sodium alginate and corn starch significantly enhances the feasibility of probiotic bacteria compared to non-encapsulated free cells [166]. The use of PVA as a material for encapsulating probiotic bacteria, including *Bifidobacterium animalis* subsp. Lactis *Streptococcus thermophilus*, and *L. paracasei*, have shown considerable improvements in viability. Encapsulating probiotic bacteria by PVA, along with the application of sodium alginate and corn starch in electro-spun nanofiber mats, has demonstrated notable improvements in viability and survival rates [166]. These findings highlight the potential of these encapsulation methods for enhancing the efficacy of probiotic delivery systems.

### 4.9. Microencapsulation by complex coacervation

Microencapsulation techniques, particularly complex coacervation, offer substantial benefits for delivering probiotics in low-pH or fermented food products. Complex coacervation encapsulates probiotics within a protective matrix composed of polymers or biopolymers, which dissolve or break down in the GI environment. This method ensures controlled release, protecting probiotics and enhancing their viability throughout digestion [167]. The encapsulation of *L. acidophilus* using complex coacervation has demonstrated increased survival in GI tract simulations, improved thermal resistance, and stability during long-term storage, underscoring its potential for food and probiotic product development [168],[169].

Complex coacervation is effective in providing a protective environment for probiotics, extending their survival in various food matrices [103]. Characterization of encapsulation materials using techniques such as TGA/DTA analysis, FTIR, SEM, and particle size distribution reveals their thermal behavior, composition, and microscopic structure, offering insights into enhancing probiotic survival under challenging conditions [170],[171]. Core-shell microgel encapsulation, using a calcium alginate core and chitosan coating, has notably improved the viability and stability of probiotics in the GI tract. This process, complemented by membrane vibration technology, facilitates the encapsulation of probiotics within the microgel structure [172]. Encapsulation of *Enterococcus durans* with alginate, Arabic gum, and psyllium beads has improved survival rates, with alginate-GA and alginate-*Phytophthora sojae* Y7 beads emerging as quick delivery carriers, showing potential for efficient probiotic distribution in functional foods and dietary supplements [173]. Biopolymer encapsulation methods with alginate, Arabic gum, and psyllium effectively protect probiotics against harsh conditions like low pH and high bile salt concentrations, ensuring their stability in various applications [173],[174]. Encapsulation of alginate particles coated with *Bacillus licheniformis* through ionic gelation aims to extend probiotic feasibility during storage and ensure successful delivery in aquaculture, addressing challenges in shrimp farming [175]. Additionally, encapsulation of *L. plantarum* 15HN with an herbal-based combination of nine gels, including alginate and psyllium, has shown significant improvements in stability and delivery under acidic and bile salt conditions, making it suitable for functional foods and supplements [176]. Encapsulation of lactobacilli and bifidobacteria with biopolymers has resulted in changes in population levels and viability under simulated GI conditions. This necessitates further research to optimize encapsulation for enhanced viability and effectiveness [177],[178]. The encapsulation of *L. plantarum* in yogurt using 3% alginate and chitosan has significantly improved survival rates during a 38-day storage study, indicating potential applications in the dairy industry for developing functional probiotic yogurt products [179]. Biopolymer encapsulation via extrusion methods has enhanced the viability and stability of probiotics, surpassing single-material encapsulation and free cells, and is promising for the nutraceutical and food sectors [180]. Single or combined biopolymer beads, including chitosan, alginate, whey protein, and carboxymethyl cellulose, have been fabricated and characterized, demonstrating efficient protection of probiotics against GI conditions, high temperatures, and long-term storage. This advancement aids in the formulation of probiotic-enriched food items with improved stability [181]. Sodium alginate capsules have proven more effective than carrageenan in yogurt matrices, enhancing probiotic viability and addressing challenges within the yogurt matrix [181]. The microencapsulation of legumes with alginate composites also offers promising prospects for delivering probiotics in supplements and foodstuffs [181]. A double-coated biopolymer, featuring layers of alginate, xanthan, gellan, and chitosan, has shown improved survival of *L. acidophilus* under GI conditions and during food processing, including bread-making [182]. Encapsulation in frozen dairy desserts provides several health benefits and presents marketing opportunities for functional food products [183],[184]. Alginate or gellan-based edible films incorporating *Bifidobacterium lactis* Bb-12 (at 10^6 CFU/g) offer potential benefits such as enhanced food stability and health benefits [185]. Casein edible films with probiotics have shown antimicrobial and antioxidant activities and improvements in structural, thermal, and optical properties [186]. The encapsulation of *L. acidophilus* with alginate, inulin, and xanthan gum effectively prevents carrot juice fermentation. Additionally, encapsulation of *Lactobacillus sakei* Bactoferm-B2 with alginate beads enhances heat tolerance in meat products [187]. Chitosan/alginate microencapsulated *L. plantarum* supplementation significantly improved natural immunity, disease protection, and growth performances in Nile tilapia [8],[28],[188].

### 4.10. Phase separation

In encapsulation and coacervation, phase separation refers to dividing a liquid mixture into distinct phases. In probiotic encapsulation, phase separation techniques such as coacervation are employed to form protective coatings around probiotic bacteria. This approach enhances the stability and viability of probiotics by shielding them from harsh GI conditions. Coacervation creates a gel-like coating that improves the survival rate of probiotics during storage and transit through the digestive system [115]. Researchers have found that encapsulation via coacervation can significantly increase probiotics' shelf life and effectiveness, demonstrating its importance in developing more effective probiotic supplements [115].

### 4.11. Sol-gel encapsulation

Sol-gel encapsulation involves embedding materials within a sol-gel matrix, converting a solution into a gel-like structure through polymerization. This method is increasingly used in probiotic encapsulation to create a protective matrix around probiotic bacteria. The sol-gel process enables the entrapment of probiotics within a stable, biocompatible matrix that can enhance their stability and controlled release. A recent study indicated that sol-gel encapsulation can improve the viability of probiotics under various storage conditions and during GI transit, making it a valuable method in the development of probiotic formulations [173].

### 4.12. Spray cooling

Spray cooling involves applying a fine mist of cooling fluid to facilitate heat dissipation. Although not traditionally associated with probiotic encapsulation, spray cooling can be relevant in the processing of probiotic powders. For instance, it can be used to cool and solidify probiotic powders quickly, thereby preserving their viability during the production process. Research has demonstrated that controlling the cooling rate during probiotic powder production can impact the final product's stability and effectiveness [189].

### 4.13. Fluidized

Fluidization refers to transforming a solid substance into a fluid-like state by suspending it in a gas or liquid. In probiotic encapsulation, fluidized bed technology is used to apply coatings or to mix probiotics with excipients effectively. Fluidized bed systems facilitate uniform coating and enhance the stability of probiotic bacteria, and the technology has been employed in the production of probiotic capsules and tablets, where it helps ensure even distribution and optimal encapsulation of probiotics, thus improving their performance and shelf life [50].

### 4.14. Air suspension coating

Air suspension coating involves suspending particles in an air stream while applying a coating material. This method is relevant to probiotic encapsulation as it allows for the uniform application of protective coatings around probiotic particles. This technique is used to produce probiotic granules with controlled-release properties, ensuring that the probiotics are protected and delivered effectively. Recent advancements in air suspension coating improve the consistency and effectiveness of probiotic formulations, enhancing their stability and viability [51].

### 4.15. Compression coating

Compression coating involves applying a coating to tablets or granules by compressing a powdered coating material onto their surface. In probiotic formulations, this method is used to create a protective layer around probiotic tablets or granules, which helps shield them from environmental factors and controlling their release and has been shown to improve the stability and controlled release of probiotics, making it a valuable approach in the development of probiotic supplements [85].

### 4.16. Co-crystallization coating

Co-crystallization coating involves forming a co-crystal with a pharmaceutical compound to create a coating on dosage forms. While not commonly used for probiotics, co-crystallization techniques can potentially be adapted to enhance the stability and solubility of probiotic formulations. Researchers are exploring the use of co-crystallization to improve the physicochemical properties of probiotic ingredients, which may lead to more effective delivery systems in the future [86],[87].

### 4.17. Cyclodextrin inclusion

Cyclodextrins are cyclic oligosaccharides that form host-guest complexes with various molecules. In probiotic encapsulation, cyclodextrins can be used to improve the solubility and stability of probiotic strains. By including probiotics in cyclodextrin complexes, it is possible to protect them from degradation and enhance their delivery to the intestine, and the technique has shown improved effectiveness of probiotic supplements by enhancing their stability and bioavailability [88],[98].

### 4.18. Rotating disk

In probiotic encapsulation, rotating disk technology can be used in processes such as coating or granulation. It helps achieve uniform application and mixing, which is crucial for producing high-quality probiotic formulations. Using rotating disks in probiotic production can improve the consistency and performance of probiotic products [99],[100].

### 4.19. Solvent evaporation

Solvent evaporation involves transitioning a solvent from a liquid to a gaseous state, leaving behind a solute or residue. In probiotic encapsulation, solvent evaporation can be used in techniques such as spray drying to produce probiotic powders. This method helps to remove solvents from the probiotic formulations, resulting in stable and effective probiotic powders, and researchers have found that optimizing solvent evaporation conditions is key to maintaining the viability and quality of probiotic powders [102].

### 4.20. Encapsulation efficiency

The encapsulation efficiency is determined by breaking down the encapsulated biopolymer bacterial element in a phosphate buffer with a pH of 7.4. Concise procedure is 50 mg of encapsulated beads is decomposed in 10 mL of phosphate buffer (pH 7.4) at 37 °C for 30 minutes. The viable bacteria captured during this process are quantified by plating on MRS agar and incubating anaerobically at 37 °C for 24 hours. Colony counts are calculated based on the number of Colony Forming Units (CFU) per gram of the product. The encapsulation efficiency for both single and combined biopolymer-coated probiotics is determined using a general formula [189]:


Encapsulation Efficiency=(log10N/log10N0)×100. (1)

Here, *N* is the figure of viable bacteria (CFU) entrapped by biopolymers, and *N*0 is the digit of free viable bacteria before encapsulation.

### 4.21. Storage stability

Recent advancements in probiotic research emphasize the importance of evaluating the storage stability of both encapsulated and non-encapsulated probiotic bacteria to ensure their viability and effectiveness where the stability of probiotic bacteria is studied over a 4-week storage period at 4 °C. Viability measurements are sampled at 1, 3, 7, 14, 21, and 28 days, and the viable percentage of probiotic cells are determined using the pour plate method, which involves plating serially diluted samples onto MRS agar plates, incubated anaerobically at 37 °C for 24 hours. This approach allows a comprehensive assessment of how encapsulation impacts the stability and viability of probiotic bacteria under refrigerated storage conditions, reflecting recent improvements in probiotic preservation techniques and methodologies [190],[191].

## 5. Characteristic features of probiotics

Probiotics exhibit a wide array of beneficial properties that make them highly valuable in both clinical and commercial applications. One of the primary characteristics of effective probiotics is their non-pathogenic nature, ensuring safety for human consumption. Moreover, their ability to withstand harsh GI conditions, such as low pH in the stomach and bile salts in the small intestine, is critical for maintaining their viability and functionality. Acid and bile salt resistance enables probiotics to survive transit through the digestive system and reach the intestines, where they exert their beneficial effects. Probiotics also can auto-aggregate and adhere to the intestinal epithelium, which enhances their colonization and strengthens the gut barrier. This promotes the competitive exclusion of pathogenic microorganisms, preventing their colonization and reducing the risk of infections [50],[192].

Furthermore, probiotics produce a variety of antimicrobial compounds, such as bacteriocins and organic acids, that inhibit the growth of pathogens. Their role in modulating the immune system is also well-documented, as they stimulate the production of immunoglobulins and cytokines, thereby enhancing gut immunity. Probiotics contribute to maintaining gut homeostasis by regulating the composition of the gut microbiota and promoting the growth of beneficial bacteria. In addition to their gut-specific effects, probiotics play a pivotal role in the development of the gut-brain axis, influencing the production of neurotransmitters like serotonin and gamma-aminobutyric acid, which are essential for mental health and cognitive function. Emerging evidence supports their potential in reducing neurotoxic effects and preventing neurological disorders such as Alzheimer's and Parkinson's diseases. These diverse characteristics underscore the importance of probiotics in maintaining overall health and preventing a range of diseases, highlighting their significant potential in future therapeutic interventions [193],[194].

## 6. Importance of encapsulation with biopolymers

Probiotics are living microorganisms that can be degraded by various environmental factors, leading to a loss of viability and effectiveness. These factors include exposure to extreme temperatures, oxygen, moisture, light, and acidic or alkaline conditions, such as those found in food processing, storage, or passage through the GI tract. Specifically, the high acidity of the stomach, digestive enzymes, and bile salts can significantly reduce the survival rate of probiotics before they reach the intestines, where they exert their beneficial effects. Additionally, oxidative stress and mechanical forces during processing or packaging can damage probiotic cells, further reducing their viability. Encapsulation with biopolymers helps protect probiotics by creating a physical barrier that shields them from these harmful conditions. This process involves enclosing the probiotic cells within a protective matrix made of biopolymers like alginate, chitosan, or gelatin. These materials provide stability and resistance to harsh environments, enabling probiotics to survive longer, remain viable during storage, and reach their target sites in the body, where they can exert their beneficial effects [56],[195],[196]. Encapsulation with biopolymers has proven to be a successful method for maintaining the stability of probiotics during storage and protecting them against favorable conditions in the digestive tract. Overall, encapsulation with biopolymers contributes to the overall effectiveness of probiotics by ensuring their survival, controlled release, and colonization potential in the gut. This approach has widespread applications in the food, pharmaceutical, and nutraceutical industries, where probiotics are utilized for health and functional purposes [196], and encapsulation with biopolymers is a crucial tool for enhancing the stability and viability of probiotic bacteria, especially when subjected to harsh conditions during food processing and storage. This innovative technique not only safeguards probiotics from environmental stresses but also ensures their controlled release and efficacy in the intended target areas, such as the GI tract. Furthermore, the acceptance of encapsulation with biopolymers and probiotics in the food production sector underscores its significance in improving the quality and functionality of food products. This approach aligns with the growing interest in incorporating probiotics into various food items to promote health and well-being [197],[198],[52]. Encapsulation by biopolymer materials possesses several characteristic features that make it a preferred choice for various applications, particularly in the food industry. Using biopolymer materials for encapsulation offers a range of advantages, including safety, sustainability, and enhanced functionality. However, careful consideration of factors like concentration, cost, and carrier materials is essential for successful implementation in various applications, particularly in the food industry [53]. Encapsulating probiotic bacterial cells with biopolymers has garnered significant attention, particularly in acidic products like yogurt, and has revealed effectiveness by increasing the viability and stability of probiotics, addressing challenges posed by the acidic environment of yogurt. This approach addresses challenges related to acidity, providing a solution that benefits both the product's stability and the overall health benefits associated with probiotics.

## 7. Biopolymer-encapsulated probiotics in biomedical applications

Combining biopolymer coatings with probiotics offers a substantial improvement in their performance under simulated GI conditions and enhances their mucosa-adhesive properties. The microencapsulation of *Ligilactobacillus salivarius* Li01 holds significant promise for enhancing probiotic viability, making it a viable option for clinical applications in the treatment of Irritable Bowel Diseases (IBD) [54]. The use of biopolymer-coated probiotics, specifically alginate-polylysine-alginate (APA) microcapsules containing strains such as *L. rhamnosus* NCIMB 6375, *L. plantarum* NCIMB 8826, and *L. fermentum* NCIMB 5221, has demonstrated significant health benefits in patients. The study suggests that APA-coated probiotic microcapsules have the potential to positively impact cardiovascular health by reducing cholesterol and triglyceride levels. Additionally, the observed improvements in gut microbiota composition highlight the broader benefits of this biopolymer-coated probiotic approach. The findings underscore the potential of such formulations as functional foods or supplements for individuals aiming to improve their cardiovascular and digestive health [55]. Utilizing microencapsulated biopolymers and probiotics extends beyond cardiovascular health and metabolic syndrome, encompassing applications in wound care and infection prevention. The controlled release and enhanced stability provided by microencapsulation techniques contribute to the observed positive effects in preclinical models. These findings suggest a multifaceted approach to utilizing microencapsulated biopolymers and probiotics in addressing different aspects of health and wellness [56]. The microencapsulation of biopolymer and probiotic cells is being explored for its potential to inhibit urogenital pathogens and prevent or treat female urogenital infections. This application suggests a targeted and controlled release of probiotics in the urogenital tract, offering specific benefits to women's health. Overall, exploring microencapsulated biopolymers and probiotics for urogenital health suggests a promising avenue for addressing specific health needs in women. Further research and clinical studies will be crucial to validate the effectiveness and safety of this approach in preventing and treating urogenital infections [57]–[59].

## 8. Conclusion

The encapsulation of probiotics using biopolymers has emerged as a critical strategy for enhancing the stability, viability, and efficacy of probiotics across diverse fields, including food, pharmaceuticals, and medicine. We discuss various encapsulation techniques such as spray drying, emulsion, extrusion, spray freeze drying, layer-by-layer assembly, electrospinning, complex coacervation, and ionic gelation. These methods, employing single or combined biopolymer materials, have significantly improved the survival of probiotics under harsh conditions such as acidic environments, bile salts, and freezing temperatures. Such advancements have enabled the targeted delivery of probiotics to the intestinal tract, the development of bio-functional foods, pathogen inhibition, and disease treatment. The versatility and effectiveness of biopolymer-based encapsulation highlight the enhanced efficacy of probiotics when combined with biopolymers, compared to unencapsulated probiotics. However, as the precise mechanisms behind the synergistic effects of biopolymer-probiotic interactions are poorly understood, further investigations are needed. This review provides a foundation for future research by emphasizing the importance of studying the synergistic relationships between biopolymers and probiotics. Researchers should focus on optimizing encapsulation techniques by exploring the combination of hydrophilic and hydrophobic properties of biopolymers and investigating the use of probiotic-based components and nanoparticles. These research areas hold promise for innovative applications in agriculture, food, and biomedicine, contributing to a more comprehensive understanding of biopolymer-probiotic interactions and expanding their potential use across multiple sectors.

## Use of AI tools declaration

The authors declare they have not used Artificial Intelligence (AI) tools in the creation of this article.
